# Division of Labor Brings Greater Benefits to Clones of *Carpobrotus edulis* in the Non-native Range: Evidence for Rapid Adaptive Evolution

**DOI:** 10.3389/fpls.2016.00349

**Published:** 2016-03-30

**Authors:** Sergio R. Roiloa, Rubén Retuerto, Josefina G. Campoy, Ana Novoa, Rodolfo Barreiro

**Affiliations:** ^1^BioCost Group, Department of Animal Biology, Plant Biology and Ecology, Faculty of Science, University of A CoruñaSpain; ^2^Unit of Ecology, Faculty of Biology, University of Santiago de CompostelaSantiago de Compostela, Spain; ^3^Centre for Invasion Biology, Department of Botany and Zoology, Stellenbosch UniversityMatieland, South Africa; ^4^Invasive Species Programme, South African National Biodiversity Institute, Kirstenbosch Research CentreClaremont, South Africa

**Keywords:** biological invasions, biomass allocation, *Carpobrotus edulis*, chlorophyll fluorescence, clonal integration, division of labor, local adaptation, spectral reflectance

## Abstract

Why some species become invasive while others do not is a central research request in biological invasions. Clonality has been suggested as an attribute that could contribute to plant invasiveness. Division of labor is an important advantage of clonal growth, and it seems reasonable to anticipate that clonal plants may intensify this clonal attribute in an invaded range because of positive selection on beneficial traits. To test this hypothesis, we collected clones of *Carpobrotus edulis* from native and invasive populations, grew pairs of connected and severed ramets in a common garden and under negative spatial covariance of nutrients and light to induce division of labor, and measured biomass allocation ratios, final biomass, and photochemical efficiency. Our results showed that both clones from the native and invaded range develop a division of labor at morphological and physiological level. However, the benefit from the division of labor was significantly higher in apical ramets from the invaded range than in ramets from the native area. This is a novel and outstanding result because it provides the first evidence that the benefit of a key clonal trait such as division of labor may have been subjected to evolutionary adaptation in the invaded range. The division of labor can therefore be considered an important trait in the invasiveness of *C. edulis.* An appropriate assessment of the influence of clonal traits in plant invasions seems key for understanding the underlying mechanisms behind biological invasions of new environments.

## Introduction

The establishment of invasive alien species modifies the stability and functioning of populations, communities, and ecosystems, displaces native species and, as consequence, promotes a loss of biodiversity (Vitousek et al., 1996; Mack et al., 2000; Strayer, 2012). In a globalized world, biological invasions and their negative impacts increased dramatically during the last decades and represent one of the most important threats to the conservation of biodiversity worldwide (Vitousek et al., 1996; Mack et al., 2000). The study of biological invasions is a rapidly developing field in modern ecology, and a crucial request in this research area is to determine the traits underlying the invasion process. However, this issue remains unsolved (Roy, 1990; Groves and di Castri, 1991; Lodge, 1993; Rejmánek and Richardson, 1996; Alpert et al., 2000; Levine et al., 2003) and more effort needs to be devoted to identify the mechanisms that explain the success of invasive species (Alpert et al., 2000; Levine et al., 2003; Blackburn et al., 2011).

It seems reasonable to assume that some plant characteristics might better explain the success of invasive species than others. In particular, clonal propagation has been suggested as an attribute that could contribute to plant invasiveness (Pyšek, 1997; Liu et al., 2006; Wang et al., 2008; Song et al., 2013). In fact, many of the most aggressive invasive plant species show clonal growth, and a recent study has highlighted the importance of traits related to clonal propagation in successful invaders (Song et al., 2013). Key aspects such as physiological integration or the capacity for a division of labor make a significant contribution to the success of clonal plants in a wide range of habitats (Hartnett and Bazzaz, 1983; Slade and Hutchings, 1987; Alpert and Stuefer, 1997; Kliměs et al., 1997; Saitoh et al., 2002; Roiloa et al., 2007). Here, we aim to add new evidence that may help to elucidate the role of clonal traits in plant invasions.

A pivotal task in plant invasions is to clarify how exotic plants adapt to the new environments that they are invading. Three mechanisms could explain the adaptation to the invaded range: (i) ‘pre-adaptation’, when species show similar trait standards in native and invaded ranges, indicating that the trait is a successful strategy in either area (Fridley and Sax, 2014); (ii) ‘phenotypic plasticity’, when responses allow plants to adjust their morphological and physiological responses very precisely to the challenges presented by particular environmental conditions, promoting resource acquisition and success in a new habitat (Grime and Mackey, 2002; Valladares et al., 2007; Mommer et al., 2011); and (iii) ‘local adaptation’, whenever there is a rapid adaptive evolution due to new selection pressures, resulting in improved fitness in the introduced environment (Maron et al., 2004; Cano et al., 2008; Xu et al., 2010; Buswell et al., 2011). Common garden experiments comparing plants from native and invaded ranges are required to test whether trait shifts are due to phenotypic plasticity or local adaptation (Wolfe et al., 2004; Erfmeier and Bruelheide, 2005; Güsewell et al., 2006; Zou et al., 2007). Trait differences between populations from native and invaded ranges grown alongside in a common garden would be indicative of an episode of local adaptation to the introduced range. This result would support the evolution of invasiveness hypothesis where rapid genetic changes are driven by natural selection pressures in the invaded environment (Lee, 2002; Stockwell et al., 2003).

One of the most striking attributes of clonal growth is the capacity for a ‘division of labor’ (i.e., specialization to acquire locally abundant resources, thereby increasing clone’s overall performance). Stolon and rhizome internodes allow resource sharing between the connected ramets of a clonal system, which therefore are physiologically integrated. Resources are generally transferred from ramets growing under conditions of high resource supply to ramets located in areas where resource supply is low, following a source-sink system (Hartnett and Bazzaz, 1983; Roiloa and Retuerto, 2005). As result of this integration, supported ramets benefit in terms of growth and survival (e.g., Hartnett and Bazzaz, 1983; Slade and Hutchings, 1987; Saitoh et al., 2002; Roiloa and Retuerto, 2006). Heterogeneous distribution of essential resources, at spatial and temporal scale, is a characteristic of many natural environments (Chapin et al., 1987; Lechowicz and Bell, 1991; Caldwell and Pearcy, 1994). In this sense, natural habitats posses both favorable and unfavorable patches which are often negatively correlated in the space (Stuefer and Hutchings, 1994). This patchy distribution of resources commonly conducts to situations in which the resource-acquiring structures of clonal plant occupy sites that differ in quality (Hutchings and Wijesinghe, 1997). When the availabilities of two essential resources are negatively correlated in space, physiological integration can induce a plastic response in which one ramet specializes to acquire the resource that is locally abundant to it but scarce to other ramets (Friedman and Alpert, 1991; Birch and Hutchings, 1994; Stuefer et al., 1996; Alpert and Stuefer, 1997). Because the resource acquisition is expected to be more economical at high concentrations, the subsequent reciprocal transfer of resources between ramets should increase the overall performance of the clone (Stuefer et al., 1996; Alpert and Stuefer, 1997; Hutchings and Wijesinghe, 1997; Stuefer, 1998). This specialization to acquire locally abundant resources is termed ‘division of labor’ (Alpert and Stuefer, 1997; Hutchings and Wijesinghe, 1997; Stuefer, 1998), and appears to be a singular trait of clonal species. In comparison, non-clonal plants or disconnected ramets in a clonal system, typically respond to resources availability by following the optimal partitioning theory that predicts an increase in the relative allocation of biomass to structures specialized in acquiring the most limiting resource (Thornley, 1972; Bloom et al., 1985).

Plant invaders usually show faster growth rates in the introduced range than in the native area (Elton, 1958; Leger and Rice, 2003; Jakobs et al., 2004; Bossdorf et al., 2005). Division of labor could be accepted as an important advantage of clonal propagation, both at the native and invaded range. However, as the invasion process can be considered as a continuum with a number of filters that the successful invader must overcome (Richardson et al., 2000), it seems reasonable to anticipate that clonal plants may intensify this clonal attribute in the invaded range given a positive selection on beneficial traits, resulting in a rapid adaptive evolution for this trait (Roiloa et al., 2015). To test this hypothesis, we (i) collected clones of *Carpobrotus edulis* from native and invasive populations, (ii) grew pairs of connected and severed ramets in a common garden experiment, under negative spatial covariance of nutrients and light, in order to induce division of labor, and (iii) measured biomass allocation ratios, final biomass, and photochemical efficiency to quantify ramet specialization and performance at morphological and physiological level. Our specific hypothesis is that the capacity for division of labor, and consequent benefit, would be greater in populations from the invaded range (i.e., Portugal and Spain) than in those from the native range (i.e., South Africa). Thus, we predict that (i) connection would induce an increase in proportional root mass and a decrease in photochemical efficiency in ramets under low light and high nutrients, and that this specialization to acquire below-ground resources will be greater in ramets from the invaded range than in those from the native range; (ii) connection would induce a decrease in proportional root mass and an increase in photochemical efficiency in ramet under high light and low nutrients, and that this specialization to acquire above-ground resources will be greater in ramets from the invaded range than in ramets from the native range; and (iii) the subsequent reciprocal transfer of resources between connected ramets should increase overall performance of the clone, and this benefit would be greater in clones from the invaded range than in clones from the native range.

Many experiments have compared relative performance in invasive and native or exotic non-invasive species (Pyšek and Richardson, 2007; Garcia-Serrano et al., 2009). Nonetheless, relatively few studies have determined whether invaders change their functional strategies from the native to the introduced range (Zou et al., 2007; Leishman et al., 2014; Heberling et al., 2015). Our study appears to be the first in testing these differences for a key clonal trait such as division of labor in an aggressive invader. Assessing the influence of clonal traits in plant invasions is key for understanding the underlying mechanisms behind biological invasions and, therefore, for predicting future invasion scenarios as well as for designing efficient control strategies in invaded areas. Additionally, this information is likewise important for a better understanding of how plants respond and evolve in new environments.

## Materials and Methods

### Study Species

*Carpobrotus edulis* (L.) N.E. Br., is a mat-forming succulent clonal plant, native to the Cape Region (South Africa), and an aggressive invader in coastal ecosystems of all the Mediterranean climate regions around the world, including Australia, Europe and America (D’Antonio and Mahall, 1991; Traveset et al., 2008; Vilà et al., 2008). *C. edulis* forms dense mats and spreads horizontally by the production of numerous apical ramets that remain physiologically integrated by stolon connections (Wisura and Glen, 1993). Ramets produce roots after direct contact with the substrate and can survive if disconnection from the parent ramet occurs. This type of vegetative growth allows *C. edulis* a very effective colonization of the surrounding area, competing aggressively with local species and affecting negatively the diversity of the native flora (D’Antonio and Mahall, 1991; Traveset et al., 2008). Previous experiments have studied several aspects of the ecology of *C. edulis* as plant–pollinator networks, plant–soil feedbacks, or hybridization studies (Vilà and D’Antonio, 1998; Bartomeus et al., 2008; de la Peña et al., 2010). However, the importance of clonal traits in the expansion of this aggressive invader has been generally overlooked (but see Roiloa et al., 2010, 2013, 2014a,b).

### Sampling Protocol

**FIGURE 1:**
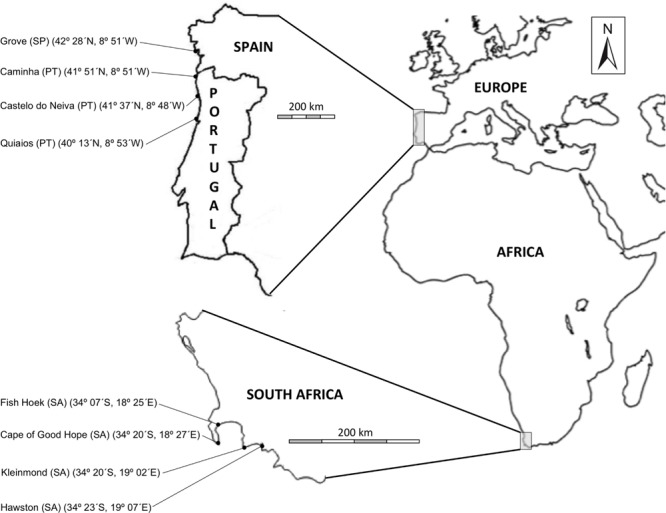
**Site location (latitude and longitude) of *Carpobrotus edulis* populations sampled from the native (Cape Region in South Africa) and the invaded (Portugal and Spain in Europe) regions.** (SA, South Africa; PT, Portugal; SP, Spain).

Four spatially separated populations of *C. edulis* were sampled in the native (Cape Region, South Africa) and four in the invaded range (Iberian Peninsula, South Europe) (see **Figure 1**). Plant material was collected in the native and in the invaded range from coastal sand dune systems where *C. edulis* typically inhabits. To have a more comprehensive illustration of the genetic variability, we selected 36 separated clumps in each of the eight populations sampled. Within a population, selected clumps were separated at least 25 m from the others. *C. edulis* forms compact clumps (Wisura and Glen, 1993) and it is reasonable to assume that each separated clump represents a different genotype. However, no genetic analyses were performed and the clumps might or might not differ in genotype. Four-member un-rooted clonal fragments were excised at the edge of each clump. Clonal fragments contained the first four units or modules (ramets *sensu* Harper, 1977) from the apices, and thus we ensured that all the experimental plant material had the same developmental stage. Plant material from the native range was collected at mid-January 2015 and maintained in the greenhouse during 3 months before the experiment began to minimize maternal environmental effects.

### Experimental Design

In April 2015, 48 ramet pairs comprised by the third and fourth ramets from the apices were selected for size uniformity from the plant stock. Initial size of ramet pairs was estimated by fresh mass. Preliminary analysis showed that the initial sizes of ramet pairs from the native and the invaded range did not differ significantly (ANOVA *F*_1,46_ = 1.787, *P* = 0.188). The experimental design consisted of two crossed factors with region (native, invaded) and connection (connected, severed) as main factors. The region factor included ramet pairs from coastal sand dunes of the native (South Africa) and invaded range (Europe), as explained above. In the connection factor, ramets within each pair were either left connected (division of labor allowed) or severed (division of labor prevented). Ramets were severed by cutting the connecting stolon halfway between them. We did not observe any immediate negative side effect of cutting the stolon (e.g., sudden death or disease). From the 36 clumps sampled in each population, we randomly selected 6 clumps to obtain the 48 ramet pairs used in the experiment (8 populations × 6 clumps). Ramet pairs from each of the eight populations sampled in the field were equally represented and randomly assigned to each combination of region by connection treatments.

**FIGURE 2:**
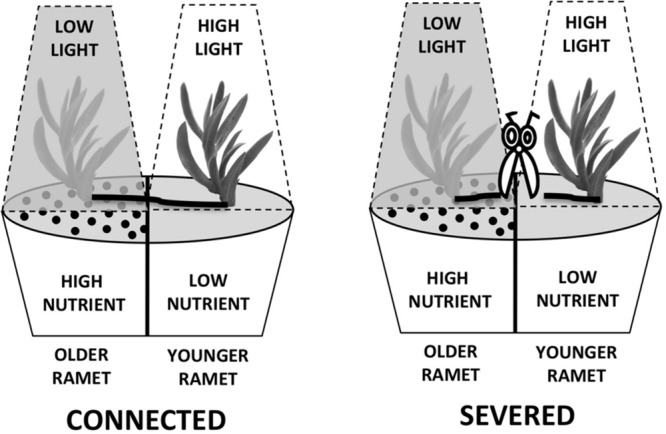
**Scheme of the experiment, showing levels of light and nutrients given to pairs of connected or severed ramets.** Older ramets were exposed to low light and high nutrient conditions. Younger ramets were exposed to high light and low nutrient conditions. See text for experimental design details.

Each pair was subjected to a regime of resource availability known to induce division of labor via changes in allocation of mass between roots and shoots (Roiloa et al., 2014b). Older ramets (the “fourth” modules) were subjected to low light and high nutrient conditions, and younger ramets (the “third” modules) were exposed to high light and low nutrients conditions (see **Figure 2**). This regime of resource availability mimics the natural conditions of *C. edulis* growing at coastal sand dune habitats, where older ramets usually grow shaded by shrubs, which enrich the nutrient content of the soil, whereas developing younger ramets spread into the non-shaded sand with low nutrient content (S. R. Roiloa, personal observation). High nutrient conditions consisted of a 3:1 mixture of potting compost and sand. In the low nutrient conditions, ramets grew in sand. Low light conditions were created using a polypropylene shade cloth that reduces ambient light to 10%. Ramets in the high light treatment were left unshaded. Each pair of ramet was planted in a single 5L plastic pot hermetically divided into two equal compartments by plastic barriers to avoid root interactions (see **Figure 2**). None of the ramets had roots at the start of the experiment. Each treatment was replicated 12 times (*n* = 12). The common garden experiment was carried out in a open-end greenhouse at the University of Santiago de Compostela (Spain) (42° 52′ 26.65″ N, 8° 33′ 31.64″ W). In order to avoid confounding effect of position within the greenhouse, the four types of treatments were interspersed randomly. Plants were watered regularly with as much water as necessary to maintain soil moisture. Treatments began on 10 April 2015 and continued for 90 days.

### Measurements

#### Spectral Reflectance

Leaf spectral reflectance parameters were measured 30, 60, and 90 days after treatment application using a portable spectrometer (UniSpec Spectral Analysis System, PP Systems, Haverhill, MA, USA). Specifically, we determined the photochemical reflectance index (PRI), that was calculated as (R_539_-R_570_)/(R_539_ + R_570_), where R_539_ and R_570_ are reflectances at 539 and 570 nm, respectively (Filella et al., 1996). This index correlates with both net CO_2_ uptake and photosynthetic radiation-use efficiency (mol CO_2_/mol photons) (Peñuelas et al., 1995; Filella et al., 1996; Gamon et al., 1997).

#### Chlorophyll Fluorescence

Immediately after reflectance measurements, chlorophyll fluorescence parameters were determined by the saturation pulse method (Schreiber et al., 1998) using a portable pulse-amplitude-modulate fluorometer (MINI-PAM photosynthesis yield analyser; Walz, Effeltrich, Germany). In particular, we measured the maximum quantum yield of photosystem II (PSII), F_v_/F_m_ = (F_m_ – F_0_)/F_m_, where F_m_ and F_0_ are the maximum and minimum fluorescence yield, respectively, of dark-adapted samples, after a saturation pulse (>5000 μmol photons m^-2^ s^-1^ of actinic white light) (see Bolhàr-Nordenkampf et al., 1989). The maximum PSII quantum yield (Fv/Fm) characterizes the photosynthetic process associated with electron transport (light reactions), and provides information on the efficiency of excitation energy capture by open PSII reaction centres (Butler and Kitajima, 1975). F_v_/F_m_ correlates with the amount of carbon gained per unit of light absorbed (Bolhàr-Nordenkampf and Öquist, 1993). This variable was measured after a 30-min dark adaptation period, which allowed the PSII reaction centers of the leaf to be fully open.

#### Growth

At the end of the experiment, each ramet was separated into shoots (including leaves and stolons) and roots, dried at 80°C for 72 h, and weighed. The total dry mass (shoot dry mass + root dry mass) and the proportional biomass allocated to roots (root-shoot ratio, RSR = root dry mass/shoot dry mass) were calculated for older and younger ramets separately. Total dry mass at whole clone level (older + younger ramets) was also calculated.

### Statistical Analysis

Prior to analyses, variables were transformed as necessary to meet the assumptions of parametric tests. Thus, the root/shoot (RSR) of older ramets were square root transformed. We analyzed differences in the total dry mass and the proportional biomass allocated to roots (RSR) by two-way analysis of variance (ANOVA) with region (native, invaded) and connection (connected or severed) as fixed effects. Separates analyses were conducted for older (with low light and high nutrients) and younger (with high light and low nutrients) ramets. Similarly, total dry mass at whole clone level (older + younger ramets) was compared by two-way ANOVA with region and connection as main factors. Changes in leaf spectral reflectance (PRI) and chlorophyll fluorescence (F_v_/F_m_) over time were analyzed with two-way analyses of variance with repeated measures (ANOVAR), using region and connection as between-subject effects. For these variables, we conduced separates analyses for older and younger ramets. Three ramets (one older and two younger) died during the experiment, and the corresponding pairs (older + younger) were excluded from the analyses. This reduced the number of replicates used in the different analyses, as indicated by the error degree of freedom. Significance level was set at *P* < 0.05. Statistical tests were performed with SPSS Statistics 19.0 (IBM, Armonk, New York, USA).

## Results

### Growth

**FIGURE 3:**
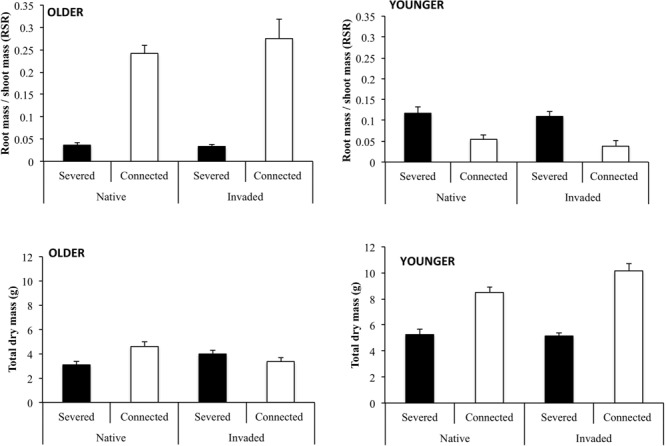
**Final measurements (mean +SE) of the proportional dry mass allocated to roots (determined as the root to shoot ratio, RSR) and the total dry mass (g) of connected and severed older **(left)** and younger **(right)** ramets from native and invaded regions.** See **Table 1** for ANOVA results.

The proportion of biomass allocated to roots by older and younger ramets, as determined by the root to shoot ratio (RSR), was significantly affected by the connection treatment (**Table 1**). Connection significantly increased the proportion of dry mass allocated to roots (RSR) in older ramets but it was decreased in younger ramets (**Figure 3**). The effect of region and the interaction between region and connection on root to shoot (RSR) was not significant neither in older nor in younger ramets (**Table 1**).

**Table 1:** Results of two-way analyses of variance (ANOVA) to examine the effects of region and connection on root to shoot ratio (RSR) of the older and younger ramets.

	Root/shoot (RSR)			Total dry mass		
Effect	df	*F*	*P*	df	*F*	*P*
Older ramet						
Region	1	0.127	0.723	1	0.318	0.576
Connection	1	163.595	**<0.001**	1	2.089	0.156
Region × connection	1	0.229	0.635	1	12.498	**0.001**
Error	41			41		
Younger ramet						
Region	1	0.898	0.349	1	3.817	0.058
Connection	1	28.124	**<0.001**	1	107.025	**<0.001**
Region × connection	1	0.122	0.729	1	4.894	**0.033**
Error	41			41		
Whole clone						
Region	nd	nd	nd	1	1.135	0.293
Connection	nd	nd	nd	1	63.965	**<0.001**
Region × connection	nd	nd	nd	1	0.111	0.740
Error	nd			41		

**FIGURE 4:**
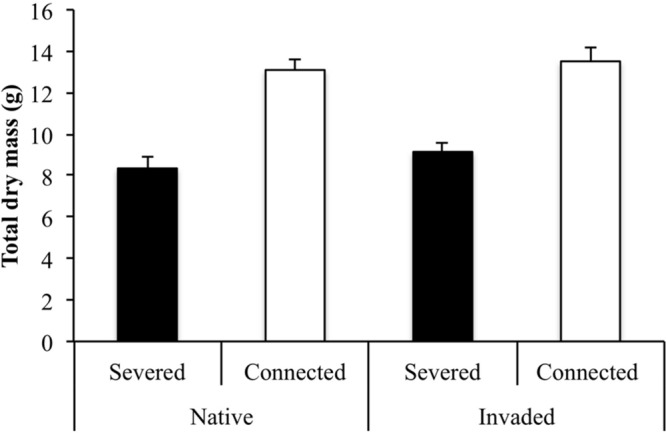
**Final measurements (mean +SE) of the total dry mass (g) of the whole clones (older + younger ramets) from the native and invaded regions in the connected and severed treatments.** See **Table 1** for ANOVA results.

*ANOVAs to detect effects of treatments on total dry mass of older and younger ramets, and at whole clone level (older + younger ramets). nd, non-determined. Values of *P* < 0.05 are in boldface. See **Figures 3** and **4** for data.*

The final total dry mass of older ramets was significantly affected by the interaction between region and habitat (**Table 1**). Connection significantly increased the total dry mass in the genotypes from the native region. However, this effect was not maintained in the genotypes from the invaded region, where we detected a decrease of the total dry mass in the connected older ramets (**Figure 3**). On the other hand, connection significantly increased the final dry mass of younger ramets (**Table 1**; **Figure 3**). This positive effect of connection on the final dry mass of younger ramets was stronger in genotypes from the invaded region, as denoted by the significant effect of the interaction between factors region and connection (**Table 1**; **Figure 3**). This result indicates that the benefit of physiological integration was significantly higher in invasive populations than in native populations. At the whole clone level (older + younger ramets), our results showed a significant effect of connection, with an increase of the final total dry mass due to connection both in the genotypes from the native and the invaded region (**Table 1**; **Figure 4**). Neither the region nor the interaction between region and connection had a significant effect on total dry mass at the whole clone level (**Table 1**).

#### Leaf Spectral Reflectance and Chlorophyll Fluorescence

**FIGURE 5:**
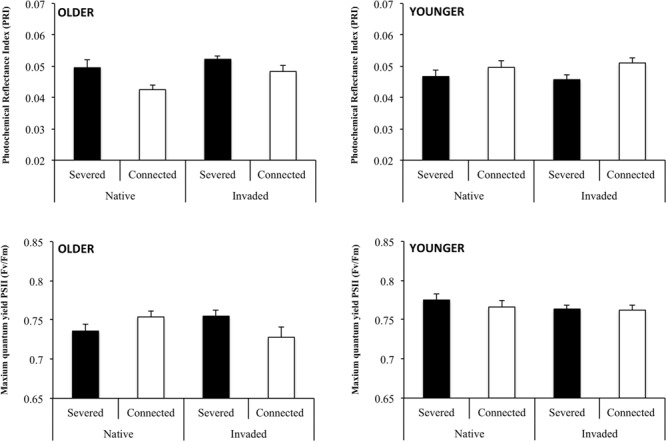
**Mean values (+SE) of photochemical reflectance index (PRI) and the maximum quantum yield of photosystem II (Fv/Fm) for connected and severed older **(left)** and younger **(right)** ramets from the native and invaded regions.** See **Table 2** for ANOVA results.

Older ramets from the invaded region showed a significantly higher PRI than older ramets from the native region (**Table 2**; **Figure 5**). Our results also showed a significant effect of the connection treatment on the PRI of older ramets (**Table 2**). Thus, there was a significant decrease in PRI of connected older ramets, regardless of whether they came from the native or the invaded region (**Figure 5**). We detected a significant effect of the interaction between region and connection in the maximum quantum yield of photosystem II (F_v_/F_m_) of older ramets (**Table 2**). Connection significantly increased F_v_/F_m_ in older ramets of the genotypes from the native region, but decreased it in the older ramets of genotypes from the invaded region (**Figure 5**).

**Table 2:** Results of two-way repeated-measure analysis of variance (ANOVAR) with region and connection as between-subject effects, for differences in the photochemical reflectance index (PRI) and the maximum quantum yield of photosystem PSII (F_v_/F_m_) of older and younger ramets.

	PRI			F_v_/F_m_		
Effect	df	*F*	*P*	df	*F*	*P*
**Older ramet**						
Between-subject effects						
Region	1	4.933	**0.032**	1	0.135	0.715
Connection	1	7.933	**0.007**	1	0.230	0.634
Region × connection	1	0.615	0.437	1	5.994	**0.019**
Error	41			41		
Within-subject effects						
Time	2	41.226	**0.001**	2	17.732	**0.001**
Region × time	2	2.154	0.123	2	1.081	0.344
Connection × time	2	0.879	0.419	2	0.012	0.988
Region × connection × time	2	0.408	0.666	2	1.049	0.355
Error	82			82		
**Younger ramet**						
Between-subject effects						
Region	1	0.008	0.928	1	1.315	0.258
Connection	1	4.635	**0.037**	1	0.579	0.451
Region × connection	1	0.397	0.532	1	0.305	0.584
Error	41			41		
Within-subject effects						
Time	2	18.015	**0.001**	2	22.316	**0.001**
Region × time	2	0.012	0.988	2	0.154	0.857
Connection × time	2	0.853	0.430	2	5.749	**0.005**
Region × connection × time	2	1.62	0.204	2	0.220	0.803
Error	82			82		

**FIGURE 6:**
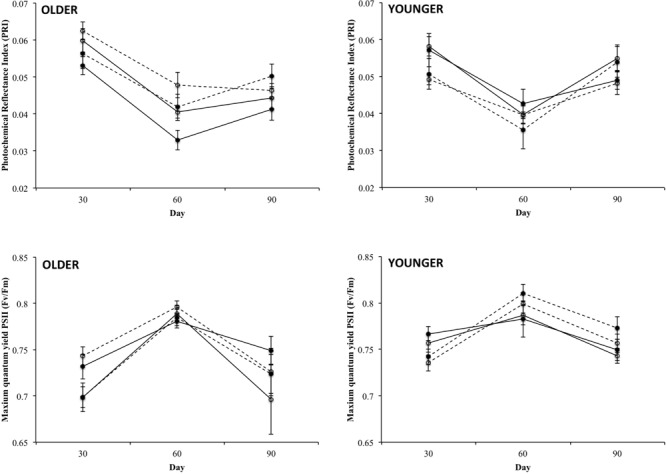
**Time course of mean values (±SE) of photochemical reflectance index (PRI) and the maximum quantum yield of photosystem II (Fv/Fm) for connected (solid lines) and severed (dashed lines) treatments of older **(left)** and younger **(right)** ramets from the native (closed symbols) and invaded (open symbols) regions.** See **Table 2** for ANOVA results.

*Values of *P* < 0.05 are in boldface. See **Figures 5** and **6** for data.*

Connection significantly affected the PRI of younger ramets (**Table 2**). PRI values were significantly higher in connected than in severed younger ramets both in genotypes from the native and the invaded region (**Figure 5**). Although we did not detect a significant between-subject effect of connection in younger ramets in terms of maximum quantum yield of photosystem II (F_v_/F_m_) (**Table 2**; **Figure 5**), this variable was significantly affected by the interaction of connection by time (within-subject effect) (**Table 2**). Thus, the F_v_/F_m_ values of younger ramets changed with time, and this change was dependent on the connection treatment. Connected and severed younger ramets significantly inverted their F_v_/F_m_ values during the experiment, regardless of their region of origin. At day 30 F_v_/F_m_ values were higher in connected than in severed younger ramets, whereas severed younger ramets showed greater F_v_/F_m_ values than connected ramets at days 60 and 90 (**Figure 6**).

## Discussion

Our results support the existence of division of labor, both at morphological and at physiological level, in native and invaded populations of *C. edulis*. As proposed in our hypotheses, we found that connection significantly increase the mass allocated to roots (RSR) in older ramets subjected to high nutrients and low light conditions, denoting a specialization to acquire the relatively abundant below-ground resource. On the other hand, connection significantly increased the photosynthetic radiation-use efficiency (as estimated by the PRI and reduced the proportional root mass (RSR) in younger ramets, which were subjected to high light and low nutrients conditions, indicating a specialization to acquire above-ground resources. In populations from both the native and the invaded range, resource sharing mediated by stolon connection significantly increased the final biomass at the whole clone level. These results provide strong evidence that ramets of clones of *C. edulis* have a capacity for division of labor, and that this specialization for acquiring the most abundant resource reports a benefit for the whole clone. However, our results do not support the prediction that clones from the invaded range may have a greater capacity for division of labor than those from the native range. Since resource acquisition is expected to be more economical where the resource is more abundant, the specialization to acquire the relatively rich resource and the subsequent reciprocal resource sharing between connected ramets would increase the overall performance of the clone (Stuefer et al., 1996; Alpert and Stuefer, 1997; Hutchings and Wijesinghe, 1997; Roiloa et al., 2014b). Similar results showing a capacity for division of labor at morphological and physiological level were recently reported by Roiloa et al. (2014b) in clones of *C. edulis* in the invaded range. Likewise, Roiloa et al. (2007) reported environmental-induced division of labor at morphological and physiological levels in clones of the stoloniferous *Fragaria chiloensis*. Both studies showed greater capacity for division of labor in clones from patchier habitats, where essential resources were negatively correlated. In these conditions, the division of labor between ramets would be specially beneficial, suggesting an adaptive division of labor induced by the environment (Roiloa et al., 2007, 2014b). Previous studies with other clonal plants also reported differences between genotypes for several clonal traits, including division of labor, resource sharing, or sexual/asexual shift, which suggest a potential for local adaptation (Lotscher and Hay, 1997; Alpert, 1999; Prati and Schmid, 2000; Alpert et al., 2003; Roiloa et al., 2007; Nilsson and D’Hertefeldt, 2008; D’Hertefeldt et al., 2014), and are in agreement with the evolutionary theory that predicts that populations evolve to generate traits to gain an advantage under their local conditions (Willians, 1966).

Although our results showed no differences in the capacity for division of labor between invasive and native populations, we found, however, significant differences in the benefits obtained by the division of labor among ramets. As reported, benefits were significantly higher in younger ramets from invasive populations than in those from native populations. This is a novel and outstanding result because it provides the first evidence that the benefit of a key clonal trait, as division of labor, may be subjected to evolutionary adaptation in the invaded range. Therefore, our results indicate that rapid genetic changes linked to selection pressures might contribute to the invasion of *C. edulis* in the introduced range. Rapid adaptive evolution of introduced populations could explain invasion success in a new environment (Maron et al., 2004; Sax et al., 2007). In this line, it seems reasonable to predict that positive selection of beneficial traits such as division of labor could be favoring the expansion of *C. edulis* in the introduced range and, therefore, promoting its invasiveness.

Interestingly, the benefit of division of labor detected in younger ramets at the invaded range seems to be obtained at the cost of the older ramets. This is, in native populations the reciprocal transport of resources between the connected older and younger ramets seems to be balanced and, consequently provided mutual benefits to both ramets. However, our results for the invasive populations showed a significant benefit of the connection for younger ramets, but a significant cost for older ramets, indicating an unbalanced share of resources. This result seemingly indicates that there was a unidirectional transport of resources from the older to the younger ramet in the population from the invaded range. As a result, growth increase was more pronounced in invasive younger ramets than in native ones, where resources transport appeared to be bidirectional, as described in previous works (e.g., Stuefer et al., 1996; Alpert and Stuefer, 1997; Hutchings and Wijesinghe, 1997). This trait shift in the introduced range could be promoting the expansion of younger ramets, contributing to the expansion of invasive plants. *C. edulis* spreads horizontally by the production of abundant younger ramets that remain integrated by stolon connections. Roiloa et al. (2010) found an association between the increase in total biomass and the horizontal expansion of apical ramets of *C. edulis* when colonizing a natural dune system in the invaded range. Thus, our finding of an intensification of the benefits derived from division of labor for apical ramets in populations from introduced range supports the idea that adaptation after introduction may favor the expansion of this aggressive invader.

Our pioneer results showing an unbalanced benefit for older and younger ramets from division of labor in populations from the invaded range could be explained within the evolution of increased competitive ability (EICA) hypothesis. The latter proposes that exotic plants, once established in the introduced range and liberated from their natural enemies, invest more in fast-growing and less in defence (Blossey and Nötzold, 1995; Mooney and Cleland, 2001; Hierro et al., 2005; Alba et al., 2012). Under this supposition, older ramets in the invasive range can reallocate resources from defensive traits to support connected younger ramets favoring the expansion of this aggressive invader. In any case, we recognize that our results are not robust enough to confirm this statement, and more experiments are necessary to elucidate if the unbalanced transport of resources described in our study could be explained by EICA hypothesis.

In addition, our results showed that photosynthetic efficiency (estimated by the PRI, which is correlated with net CO_2_ uptake and photosynthetic radiation-use efficiency, Peñuelas et al., 1995; Filella et al., 1996; Gamon et al., 1997) was significantly higher in older ramets from the invaded range than in older ramets from the native area. Previous studies have detected that invasive plants show greater growth rates in their introduced range than in the native range (Elton, 1958; Leger and Rice, 2003; Jakobs et al., 2004; Bossdorf et al., 2005). In another common garden experiment, Zou et al. (2007) also found that invasive populations of *Sapium sebiferum* showed significantly higher photosynthetic activity, in terms of net CO_2_ assimilation (determined directly by gas exchange measurements), than populations from their native range. Eco-physiological results also showed a significant increase in the PRI in connected younger ramets, both in the native and invaded range. This increases in photochemical efficiency was translated into a significant increase in the total mass, denoting a positive correlation between PRI and growth. However, and differing to the pattern obtained for total mass, we did not detect a more accentuate increase in photochemical activity in younger ramets from invaded range in comparison with values obtained in native populations. Probably, the significantly higher benefit in younger ramets from invasive populations was due to the support received from their connected older ramets. In this sense, the unidirectional transport of resources from the older to the younger ramet in the population from the invaded range increased more pronounced the growth in invasive younger ramets than in native ones, in spite of not showing differences in photochemical activity.

## Conclusion

This is the first study reporting differences between native and invaded populations in the benefits derived from a key clonal trait such as division of labor. The results from this study seem consistent with a rapid adaptive evolution of the clonar invader *C. edulis* with a positive selection of the benefits from division of labor in the invaded range. The benefits from division of labor could therefore be considered an important trait in the invasiveness of *C. edulis.* However, whether this is the case for all clonal plant invaders or not remains to be clarified, and future research should include more clonal species. The contribution of clonal traits to the capacity to establish in new environments represents an exciting research field for understanding the mechanisms underlying plant invasions, and for a better knowledge of how plants respond and evolve in new habitat. In addition, experiments testing for differences between invasive and exotic non-invasive species are mandatory to understand the role of clonal traits in plant invasions. Understanding the influence of clonal life-history traits in plant invasions seems key for predicting future invasion scenarios and for devising efficient strategies of control and restoration of invaded areas.

## Author Contributions

SR was involved in designing the experiment, field samples collection, set up the experiment, data collection, and analysis, manuscript preparation and submission. RR field samples collection, set up the experiment, data collection, and co-prepared manuscript. JC set up the experiment, data collection and co-prepared manuscript. AN and RB field samples collection and co-prepared manuscript.

## Conflict of Interest Statement

The authors declare that the research was conducted in the absence of any commercial or financial relationships that could be construed as a potential conflict of interest.
